# Are European cities achieving emission reduction commitments? A comparative analysis under the Covenant of Mayors initiative

**DOI:** 10.1016/j.heliyon.2023.e23423

**Published:** 2023-12-09

**Authors:** Benedetta Lucchitta, Valentina Palermo, Giulia Melica, Tania Molteni, Andrea Burro, Paolo Bertoldi, Edoardo Croci

**Affiliations:** a GREEN Research Center, Bocconi University, Milan, Italy; b European Commission, Joint Research Centre (JRC), Ispra, Italy

## 1 Introduction

Climate change (CC) is shaping the political and scientific debate at the global level with increasing urgency. It is clear that this challenge requires the contribution of all actors in different fields, guises, and levels of governance [[1], [2], [3], [4]]. Along with international and national commitments, local governments are involved in implementing specific actions to tackle CC, as cities are widely recognized as responsible for Greenhouse Gas (GHG) emissions while being affected by CC. The potential of cities in contributing to the climate challenge has been clearly recognized and supported within Europe by the Covenant of Mayors (CoM) initiative. Aligned with many European Union (EU) priorities and efforts, particularly the EU energy and climate targets for 2020 and 2030, the CoM is the first pan-European initiative involving local authorities enabling different levels of government to work together and complement their actions in their policy areas to contribute to a low carbon transition [5].

The CoM was launched in 2008 by the 10.13039/501100000780European Commission (10.13039/501100000780EC) to support municipalities in achieving the European 20 % CO_2_eq reduction goal by 2020 (CoM 2020). Cities' commitment is translated into Sustainable Energy Action Plans (SEAPs), primarily drawing on the findings from the Baseline Emission Inventory (BEI) and defining concrete measures for reducing emissions, translating the long-term strategy into action. Reporting on the SEAP and its implementation is also a commitment undertaken by CoM signatories. In addition to data reported in the BEIs, signatories have to report their Monitoring Emission Inventories (MEI), at least every 4 years. In 2021, the initiative (which now entails a commitment to tackle mitigation, adaptation and energy poverty) counts more than 10,500 signatories, of which 9,755 are from EU 27 [6]. Concerning mitigation, 5,763 climate action plans with a target for 2020 have been submitted by EU 27 local authorities through the MyCovenant reporting platform by May 2021. The CoM, by providing a harmonized framework and methodological guidelines for signatories to set their targets and develop their plans [7], allowed collecting a complete set of information at the local level in terms of energy consumption, carbon emissions, and adopted policies, thereby offering a large database on cities’ action to mitigate CC i.e. 8. Moreover, thanks to the significant participation of cities across Europe, the CoM forms a sort of “hub” of the variety of policies and practices cities have been implementing to address the energy and climate challenges.

As a matter of fact, while sharing a common mitigation goal, CC strategies at the local level are site-specific and suit local communities and territories’ needs and peculiarities. In addition, several drivers can influence the impacts of CC mitigation policies at the urban scale, i.e. population, urban density, Gross Domestic Product (GDP), climatic conditions, etc. The SEAPs are the instrument through which signatories express their approaches to deliver the mitigation objectives. Through their assessment, the overall policy impacts and progresses of the initiative can be tracked, and the most effective measures, sectors and instruments as well as the directions undertaken by signatories can be analysed. Some studies have explored potential leverages and influences on CO_2_ emissions reduction by cities.

[9] statistically investigated potential institutional, socio-economic, and environmental factors on local plans. The analysis showed how factors such as membership in climate networks, population, and GDP per capita act as drivers for developing mitigation and adaptation plans. [10] showed that the significant driver for intended emission reductions was the level of emissions in the base year. Cities with higher emissions were associated with higher intended emission reductions, compared to cities with lower emissions in the inventory year. Similar results were yielded by [11], showing that city emissions reduction is influenced by plan-level, city-level, and country-level characteristics and that cities with higher baseline emissions per capita reached higher emission reductions, particularly related to high-emitting sectors such as buildings and transport. [12] showed that the population has a stronger influence on the type of planned and implemented measures to reach the target, than climatic conditions and GDP. The influence of population in successful climate mitigation actions has been also shown by [13] while evaluating the barriers and drivers of cities' progress monitoring. [14] highlighted that internal factors impact policies' selection. Finally, city size and geographical location as well as cities' membership of climate networks have been significantly and positively related to cities’ mitigation ambition by [15]. Hence, the CoM shows how cities are capable to positively impact on climate by adopting a mix of policy instruments [16,17]. Recent figures from the CoM assessment report [6] show that EU-27 signatories with a 2020 mitigation commitment are well on track to achieve their collective 2020 emission reduction target. The overall committed reduction is 26.4 % by 2020 with reference to base year emissions of 797.9 MtCO_2_eq.

However, despite the positive trends, some challenges remain. Cities may lack the authority to completely transform the energy system, especially for causing a wholesale switch to renewable energy for decarbonization [13,18]. Cities have limited legislative authority on some sectors thus emissions reduction related to specific sectors derive - or are highly influenced - from policies defined at the regional or national level. This is pivotal when cities commit to more ambitious emissions reduction targets such as in the case of carbon neutrality [18,19]. The decisions on strategies and policies by cities can be assessed to understand the drivers that lead to more effective and successful outcomes. This paper follows the pathway set by former studies with the aim to understand potential factors positively or negatively influencing the results of planned measures by CoM signatories in their SEAPs by mainly focusing on policy instruments and sectors, in addition to other previously explored characteristics. To this aim, this study provides a regression analysis based on data regarding BEIs and MEIs from a sample of CoM signatories as of August 2019.

## 2 Methodology

### 2.1 Dataset structure: cities, measures and policy instruments classification

The dataset includes data submitted by CoM signatories having officially reported their MEI through the MyCovenant platform by 2019. The following criteria have been applied to gain a consistent and homogeneous sample: i) only EU 27 and United Kingdom (UK) local administrative units (LAU) have been included; and ii) the cases where emissions over time increased/did not change were excluded to focus only on the reduction's effect. The final sample is composed of 1,354 CoM EU signatories - accounting for a total of 45,636 mitigation actions reported in SEAPs - from EU 27 and UK. Signatories are distributed by country and by the number of inhabitants according to the following size categories: i) XXS <10,000; ii) XS 10,001–50,000; iii) S 50,001–100,000; iv) M 100,001–500,000; v) L 500,001 - 1,000,000; vi) XL > 1,000,000. The distribution of cities in the mentioned categories is not uniform as the “XXS” and “XS” are the most represented, which is aligned to the overall CoM trend showing 90 % of signatories with less than 50,000 inhabitants [13]. In particular, Italy and Spain show the highest number of signatories in these categories. On the contrary, Germany, France, and the United Kingdom have a larger share of cities with more than 100,000 inhabitants.

The absolute CO_2_ emissions reduction is linked to size/population of cities. Overall, the signatories in the sample have reported 476 MtCO_2_eq in their BEIs and 363 MtCO_2_eq in their MEIs, corresponding to a reduction of 113 MtCO_2_eq (24 %). While this result is in line with the target of 28 % CO_2_eq emission reduction achieved by cities by 2020,^1^ compared to BEIs for all cities having presented a MEI by August 2019 [20], the emissions reduction is not proportional to the frequency of signatories. For example, Spain is accountable for 18.8 MtCO_2eq_ emission reduction, while Italy for 16.5 MtCO_2_eq (respectively 16 % and 14 % of the sample). On the other hand, German cities represent only 2 % of the sample but account for almost 38 % of the reduction, France and the UK with 1.1 % and 0.8 % of the sample, account for, respectively, 4.04 % and 4.08 % of the reductions.

In alignment with the International Panel on Climate Change (IPCC) [21], within the CoM methodological approach, signatories report in their inventories, emissions occurring from different sectors. In CoM Europe four sectors (Municipal buildings, equipment/facilities; tertiary (non-municipal) buildings, equipment/facilities; residential buildings; and transport) are identified as "key sectors" in which local authorities can influence energy consumption and consequently reduce related CO_2_eq emissions. When reporting the actions planned in their SEAPs, signatories identify for each action the sector, the area of intervention, and the policy instruments to which the action belongs. In this paper, the actions included in the SEAPs have been classified by sector (Table 1), underlying policy instrument, and timeframe.

**Table 1:** CoM sectors.

B1 (Buildings)	B5 (Local electricity production)
B2 (Public lighting)	B6 (Local heat/cold production)
B3 (Industry)	B7 (Other)
B4 (Transport)

**Table 2:** Categories of policy instruments used in the analysis.

Category of policy instruments	Instruments included in CoM classification
Economic	Energy & carbon taxes; Road pricing; Grants & subsidies; Third-party financing
Information, dissemination, and awareness-raising	Awareness-raising & training; Energy certification & labeling
Command and Control	Energy supplier obligations; Building standards; Energy performance standards; Land use planning; Land use planning regulation; Mobility planning regulation
Energy and Environmental Management	Energy management; Green Public procurement; Integrated ticketing and charging (transport)
Voluntary Agreements	Voluntary agreement with stakeholders
Not Applicable	Actions that CoM municipalities have not categorised using policy instruments
Other	Policy instruments not mentioned in the other categories/classification and reclassified policies due to changes in the reporting requirements.

The CoM policy instruments classification has been reorganized into broader categories (Table 2). Policy instruments are usually categorised into legislative-regulatory, economic-fiscal, agreement-based, and information/communication-based, even if further categories may be applied 22. In this paper, an additional category is foreseen, related to energy and environmental management, as this is one of the main instruments adopted by local governments in managing and addressing sustainable energy issues. Furthermore, voluntary agreements have been identified as a separate category.

Finally, the intended implementation timeline of the actions has been investigated for each sector and for each category of policy instrument. Actions within each sector and category of policy instrument have been aggregated per “start” and “end” years. The analysis of the cumulative share of actions started or completed in certain years enables a better understanding of the actual progress made by local authorities in terms of GHG emissions reduction. The average duration of actions in each sector has also been calculated, to gain insights into which sectors seem to require actions with longer implementation timeframes compared to other sectors where actions can be decided and implemented faster. Results are showed and discussed in sections 3, 4.

### 2.2 Regression analysis model

Regression analysis has been widely used in literature to understand the main drivers of emissions at the urban scale [10,[23], [24], [25]] or to assess the policies' impacts to improve energy efficiency [20,26]. The results of energy policies are influenced by a variety of factors that act simultaneously, such as market forces, technological change, behaviours, and assessing the effectiveness of such policies is a complex task. Econometric methods such as regression analysis can be used to investigate the contribution of energy policies to the reduction of energy demand and related GHG emissions, disentangling the effects of different factors [26]. This approach can be particularly appropriate also for the analysis of cities’ climate policies effectiveness, as in the literature there is a lack of empirical studies on actual results achieved by cities through the implementation of their climate mitigation and emission reduction plans [27].

An Ordinary Least Squares (OLS) regression is used to identify the impact of the covariates on the reduction of the CO_2_eq emissions from the BEI year to the MEI year. This model has been selected due to the elevate accuracy of the regression coefficients. In particular, OLS is considered as one of the most effective techniques to identify the correlation among variables [28,29]. In addition to contextual data [12], our reference model explicitly includes a set of variables representing the number and variety of CO_2_ reduction policies adopted by CoM cities in their SEAPs. Such variables measure the distribution of actions among sectors and among policy levers, describing the overall policy mix and CO_2_ emission reduction strategy chosen by CoM cities throughout their plans.

The reference model is:

$$l_{delta_{BEI_{MEI_{i\;}}}}=\beta_{0}+\beta_{1}actionB1_{i}+\cdots +\beta_{7}actionB7_{i}+\beta_{8}action\_Economic_{i}+\cdots +\beta_{14}action\_Other_{i}+\alpha X_{i}+\epsilon_{i}$$

Where.

● l_delta_BEI_MEI: logarithm of the variation in the emissions from the BEI year to the MEI year;
● actionB1: number of actions in the “Buildings” sector;
● actionB2: number of actions in the “Public lighting” sector;
● actionB3: number of actions in the “Industry” sector;
● actionB4: number of actions in the “Transports” sector;
● actionB5: number of actions in the “Local electricity production” sector;
● actionB6: number of actions in the “Local heat/cold production” sector;
● actionB7: number of actions in the “Other” sector;
● action_Economic: number of actions in the “Economic” policy instrument category;
● action_Information: number of actions in the “Information, dissemination and awareness-raising” category;
● action_Command_Control: number of actions in the “Command and control” category;
● action_Energy_Management: number of actions in the “Energy and environmental management” category;
● action_Voluntary_Agreements: number of actions in the “Voluntary agreements” category;
● action_Not_Applicable: number of actions in the “Not applicable” category;
● action_Other: number of actions in the “Other” category.

**X** contains the following variables.

● delta_MEI_BEI_year: number of years from the BEI year to the MEI year;
● MEI__pop: population in the MEI year at the city level;
● Geographical location: 4 geographical dummies^2^ ;
● hdd_MEI: measure of heating degree days in the MEI year;
● MEI_gdp: Gross Domestic Product per capita in the MEI year at the NUTS 3 level;
● GDP_2: squared value of MEI_gdp^3^ ;
● density: population density in the MEI year (NUTS 3 level)

Data on the variables has been collected from different sources, reported in Table 3 below.

**Table 3:** Data used for regression variables.

Variable	Level	Source
Actions (number and type)	Local level (LAU^4^ )	CoM dataset
CO_2_eq emissions	Local level (LAU)	CoM dataset
Population	Local level (LAU)	CoM dataset
Area	Local level (LAU)	CoM dataset
HDD	Province and regional level (NUTS 2/NUTS 3^5^)	[30] EUROSTAT (2019) – NUTS 3 with the exception of Valverde (Spain) at NUTS 2. Data for the UK retrieved from Safety Central at NUTS 2
GDP	Province and regional level (NUTS 2/NUTS 3)	[31] OECD (In millions of USD, constant prices, and constant PPP, using 2015 as base year)
Density	Province level (NUTS 3)	[31] OECD: Data for the cities in Cyprus are from the World Bank and at the national level.

The units of analysis *i* are the cities included in the sample. Due to collinearity problems, one of the 4 geographical dummies has been dropped from the model, therefore, the interpretation of the coefficient of the 3 geographical dummies in the regression is the average value of the dependent variable compared to the omitted dummy. The coefficients of the regression are constructed by minimizing the sum of the squares of the residuals. The residuals are defined as the differences between the dependent variable and the fitted values, which are predicted by the model. A Breusch-Pagan test is conducted to check the presence of homoskedasticity in the error terms of the model. The null hypothesis of the test is that the error terms are homoscedastic (i.e., they have constant variance). Since the test statistic of the test in the model has a p-value (0.0010) lower than 0.05, the null hypothesis is rejected at a 5 % significance level. In this case, the conclusion is that the model suffers from heteroskedasticity. Hence, to overcome this problem and identify precise estimates, robust standard errors are used in the regression. Moreover, the logarithm transformation of the dependent variable is used to reduce the outliers’ effect.

The other covariates were not transformed; therefore, the coefficients of the regression should be interpreted as the percentage change in the reduction of emissions associated with a unitary increase of the independent variable, all else equal. Moreover, a positive (and statistically significant) coefficient should be associated with a variable that is effective in reducing emissions. In particular, since the dependent variable is the (logarithm of the) reduction of the emissions, a positive coefficient signals that the independent variable increases the gap between emissions in the BEI year and in the MEI year (all else equal). An increase in the gap could be associated with a lower level of MEI emissions, therefore, the covariate with a positive and statistically significant coefficient is able to reduce the emissions. Moreover, since the dependent variable is the logarithm of the gap, the interpretation of the coefficient is the following: a percentage variation in the gap associated with a unitary increase in the covariate, all else equal. Finally, the model could suffer from problems of endogeneity. In particular, some variables among the covariates may be correlated with the error term. The concern for endogenous regressors is due to the possible presence of the phenomenon of reverse causality. Reverse causality refers to the situation in which it is not the independent variable that has an effect on the dependent variable but is the dependent variable that affects the independent variable. In this analysis, the goal is to detect the effectiveness of the actions implemented by cities to reduce emissions. However, it could be plausible that cities with higher emissions are also the ones that implement a higher number of actions to reduce them. However, a causal interpretation of the regression coefficients is beyond the purpose of this research.

## 3 Regression analysis results

**Fig. 1:**
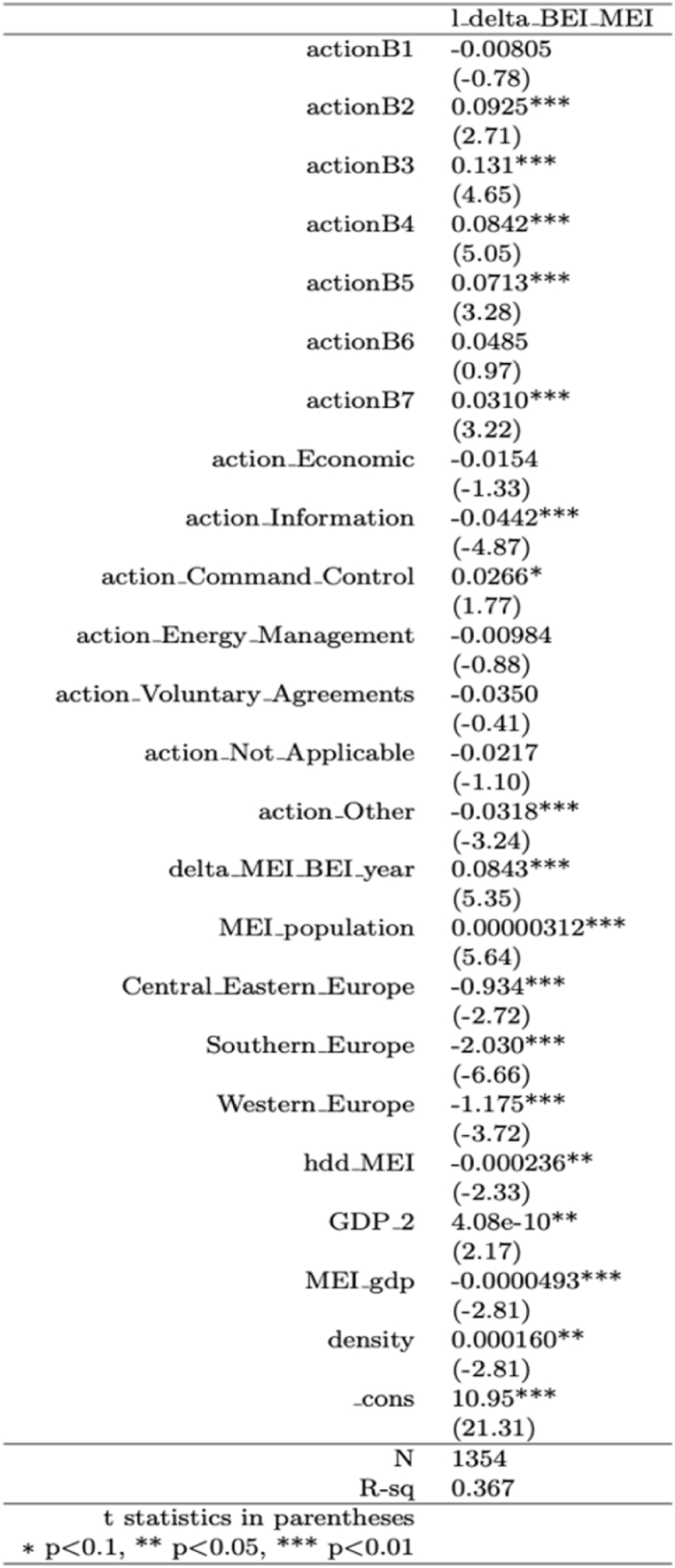
Regression analysis results.

Fig. 1 shows the regression analysis results. The model has a relatively high R-squared (0.367), also compared with other studies in the field [10,11], meaning that it is able to explain more than 35 % of the variance of the dependent variable. Results highlight that not all variables included in the regression are significant for the variation in the emissions from the BEI year to the MEI year.

The sectors whose actions are effective in reducing the emissions are: *Public lighting*, *Industry*, *Transport*, *Local electricity production*, and *Other*. From the positive and statistically significant coefficients associated with these variables, it can be concluded that almost all the sectors were effective in urban emissions reduction. By looking at the magnitude of the coefficients in the regression model, it can be inferred that the most effective sectors are *Industry* and *Public lighting*: if the number of actions in one of these sectors increases, the reduction of emissions could exceed 10 %. The other sectors are all associated with a reduction of almost 9 %, with the exception of the sector *other* which is associated with a reduction of 3 %. The only sectors without a significant coefficient are *Buildings* and *Local heat/cold production*.

Three variables related to policy instruments present significant coefficients: *Information, dissemination and awareness-raising*, *Command and control*, and *Other.* However, *Command and control* is the only policy instrument that is effective in reducing emissions, according to the model, while the coefficients of the variables that refer to the categories *Other* and *Information, dissemination, and awareness-raising* are negative. Finally, the actions without a significant coefficient are those that refer to the instruments *Economic*, *Energy and environmental management*, *Voluntary agreements*, and *Not applicable*.

The other variables with positive and statistically significant coefficients are the number of years from BEI to MEI, population, and density. On the other hand, hdd_MEI presents a negative and statistically significant coefficient. The coefficient of *MEI_gdp* is significant and smaller than 0, while the coefficient of *GDP_2* is significant and positive. Finally, since the dummy for Northern European cities has been omitted by the model to avoid collinearity, the interpretation of the coefficients of *Southern_Europe*, *Central_Eastern_Europe,* and *Western_Europe* is that cities from these areas are less effective in reducing the emissions compared to Northern European cities.

**Fig. 2:**
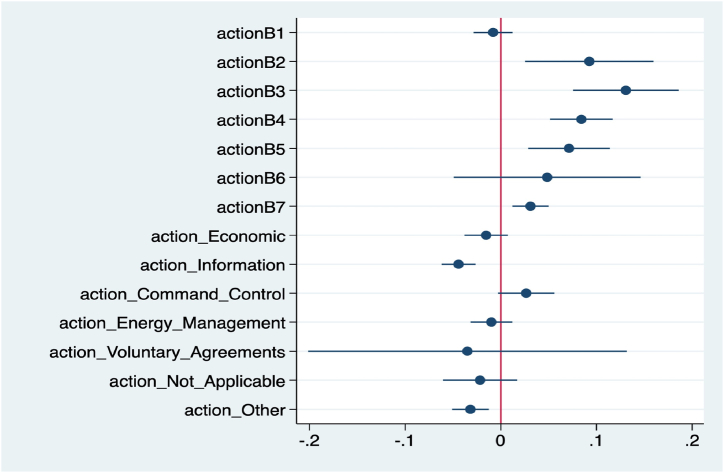
confidence intervals of regression model coefficients.

The estimates of the coefficients of the regression model with their confidence intervals are shown in Fig. 2. In particular, it plots whether the estimates of the coefficients significantly differ from 0 or not. The points represent the estimates of the coefficients, while the confidence intervals are displayed to the right and to the left of the estimates. If the confidence intervals do not cross the red vertical line, then it is possible to infer that the associated coefficient is statistically significant at a 5 % level.

## 4 Discussion

Results show that overall policies are contributing to reducing CO_2_eq emissions with differences in terms of sectors and policy instruments. Related to these elements, this section gives insights into the enabling factors influencing the reduction potential of actions.

In *public lighting*, relatively few actions were planned (6 % of total analysed actions) with a low level of reduction (2 % of total expected carbon emission reduction) (Figure a – Appendix I). Despite this, the regression shows that public lighting actions have been effective in reducing emissions. This result might be related to the use of LED technologies enabling relevant savings in this sector with relatively short payback times [32]. Furthermore, if we consider the implementation timeline of actions, public lighting has a relevant share of actions completed by 2015 (29.8 %) compared with other sectors (Table a – Appendix I), which might explain why the reductions from this sector are visible in the cities’ MEIs.

*Industry* is not among the mandatory sectors of the initiative; hence, cities tend to report only important effective actions. This is confirmed by the number of industry actions in the sample, which represent 2 % of total actions, and expected emission reductions which amount to 5 % of the total (Figure a - Appendix I). Moreover, 40 % of the actions in this sector are targeting energy efficiency in industrial processes, which implies improving processes and management and reducing related emissions. This approach is sometimes coupled with economic policy instruments, as the case of Cordoba (Spain), whose SEAP foresees a 5 % fiscal reduction for companies reducing their energy consumption through energy efficiency by at least 7 % [33]. In such a highly competitive private sector, companies plan investments that can quickly lead to significant energy and financial savings and, therefore, the planned actions in SEAPs are aiming to have substantial reductions.

The *Other* sector includes 20 % of total planned actions and is accountable for 27 % of the reductions (Figure a – Appendix I). Despite its relevance in percentage terms, the sector shows a positive coefficient from the regression results, slightly contributing to emissions reduction. In principle, actions that do not fall under the stated sectors of the initiative are included in *Other*. However, it is also possible to identify cases where the action could easily fit specific sectors and/or relate to a specific instrument. This is a consequence of the changes in the initiative's reporting structure in different timeframes^6^ and, therefore, it is not possible to fully interpret the correlation of actions within this sector.

The *transport* sector comprises 16 % of the actions in the sample, which are accountable for 23 % of the total reductions (Figure a – Appendix I). Based on the regression, the *transport* sector is effective in reducing emissions. The significant share of expected emissions reduction in this sector and the effectiveness of its actions might be linked to the high variability of instruments selected and to the larger weight attributed to *Command and control* type, which is the only policy instrument showing a positive coefficient (Figure b – Appendix I). In this category, it is common to limit the access to private vehicles towards pedestrianization. Moreover, actions in this sector often refer to the provision and upgrade of new public transit infrastructures and consider the integration of tickets, thereby resulting in significant reduction potential through modal shift and inter-modality. As an example, this is the case of the city of Koprivnica (Croatia) where the implementation of electric buses has also been the occasion to develop an integrated ticketing system for regional road and rail transport [34].

The *building* sector shows a high share in the total number of actions being responsible for 26 % of the total expected reduction (Figure a – Appendix I) while having a negative coefficient in the regression analysis. This result might be influenced by several factors, including: i) the action distribution patterns observed in the building subsectors (municipal, residential, tertiary); ii) the typology of policy instrument used to implement actions and iii) the long implementation timelines of actions. Given the relevance of buildings on total EU energy consumption and CO_2_ emissions, a specific analysis has been conducted and these three influencing factors were examined more in detail.

Considering the first factor, i), CoM local authorities are encouraged to take an exemplary role in municipal buildings. Since this building segment falls under their direct competence and is highly visible, local authorities are expected to demonstrate an ambitious yet credible path towards decarbonization. This path generally translates into a high number of planned actions, which, however, do not necessarily imply high reductions in absolute terms, given that the municipal buildings' sub-sector only accounts for a small share of a city's emissions, and consequently for a small share of estimated reductions (see Table b – Appendix I). Residential and tertiary buildings account for almost 20 % of the total planned actions, and about 23 % of the estimated CO_2_eq reduction. However, while cities and local authorities have competence in residential and tertiary buildings, the portfolio of mitigation actions and policy instruments they can deploy in those sub-sectors is more limited.

With reference to the factor ii), local authorities in the CoM context largely rely on information, dissemination, and awareness-raising measures and energy & environmental management in buildings (Figure b, Figure c, Appendix I). However, these measures alone might not be sufficient to trigger significant energy renovation interventions unless they are combined with financial instruments (such as grants or subsidies), as also shown in the regression analysis. At EU level, the annual energy renovation rate of buildings is close to 1 % (EU Renovation Wave [35]) and buildings’ decarbonization is behind the path to climate neutrality by 2050 [36]. The regression results may suggest a slow pace at improving the energy performance of the building stock. As far as new buildings are concerned, typical measures in SEAPs include building codes (about 8 % of measures on residential buildings and 6 % of measures on tertiary buildings), with cities applying more stringent energy performance requirements than national ones. However, new buildings represent a very low share of the building stock (for example the annual share of new dwellings from the total stock of permanently occupied dwellings is less than 1 % for all the years from 2010 to 2019) 7, hence, the impact of this instrument might be rather low.

In addition, considering factor iii), measures targeting municipal buildings have a shorter implementation timeframe compared to the ones targeting private buildings, as the latter requires the mobilization of various stakeholders and private investments. In the sample, about 17 % of measures on residential buildings and only 10 % of measures on tertiary buildings were expected to be completed by 2015. Hence, measures in those sub-sectors will require a longer period before their actual impacts are reflected in cities’ MEIs (Table a - Appendix I).

In the l*ocal heat/cold production* sector, the most used instruments are *Economic* (26 %) and *Other* (42 %), which according to the output of the regression, are not very effective in reducing emissions. Furthermore, measures targeting this sector represent only 2 % of the sample. However, actions in this sector show a relatively larger emission reduction potential, as they represent 10 % of expected reductions (Figure a – Appendix I). The effectiveness of local heat production actions might be due to the short implementation duration (as building new plants or distribution networks requires a defined, not long timeframe), and the consequent high share of completed actions. *Local heat production* is, in fact, the only sector with over 30 % of completed actions by 2015 (32.2 %) (Table a – Appendix I).

If the reduction potential of policy levers is considered independently of the sector, only measures categorised in the C*ommand and control* and *Economic instruments* provide significant estimated CO_2_eq reduction, although forming 23 % of the sample (Figure c – Appendix I). This confirms the increased adoption of economic instruments for tackling environmental and energy aspects at the local level, in alignment with the trends of the last decades (already [37]). In addition, local authorities are overcoming single approaches towards combining command and control and economic policy instruments [38] to deal with the complexity and context-based characteristics of environmental and energy issues at the local level. As an example, the city of Merlino (Italy) has adopted measures in the building sector responding to criteria of regulation/facilitation. The city included in its SEAP the revision of the Building Regulation with the aim to integrate energy efficiency principles in buildings [39]. While volumetric incentives for buildings with a good energy performance are already implemented, the city is striving to enforce higher standards for new buildings.

The regression developed in this study showed that regulative approaches tend to be more effective. The implicit need for an integrated approach can be also read from the high number of policies within the class of *Command and Control* referring to Urban Regeneration (18.7 %). This refers to a deep transformation of areas, usually in decline, to improve their economic, physical, social, and environmental actions, through, in this context, a sustainability lens [[40], [41], [42]]. Therefore, policies related to urban regeneration explore low-carbon and energy-efficient options in different sectors while looking at broader links with social and institutional dimensions.

Additional factors beyond the control of policymakers have been investigated. Based on the regression results the variables population size, delta_MEI_BEI_year, density, and GDP are effective in reducing emissions, while the variable HDD showed a negative coefficient.

Larger cities have often at disposal more resources to address CC mitigation planning [43], therefore, they could be able to develop a better set of mitigation actions. Furthermore, larger cities could benefit from agglomeration economies [44], which could influence the effectiveness of some low-carbon actions. This result has controversial outcomes in literature, as some studies find a relationship between population and emission reductions [12,45] while others do not find a strong association between population and emissions reductions obtained by city policies [11]. Likewise, the positive coefficient of the population density variable might imply that more densely populated cities are more effective in reducing emissions. Wide literature confirms a positive association between the two variables, as an effect of the potential choice of denser cities to invest in low-carbon infrastructure [46]. However, higher carbon reductions can be reached by actions implemented by local authorities compared to the effects gained by these two variables.

The coefficient of MEI_gdp is significant and lower than 0, while the coefficient of GDP_2 is significant and positive. This result is in line with the literature on the environmental Kuznets curve, which predicts an inverse U-shaped relationship between economic growth and environmental degradation. This means that a higher GDP has a negative impact on emissions reduction up to a certain level. Once a region has reached a certain level of development, the increase in GDP could be used to promote more sustainable projects and actions. In this study, the turning point is identified at 61 billion USD hence, for lower GDP values there is a positive relationship between economic growth and emissions, while for values above that threshold, the relationship is negative.

The relationship between GDP and more effective emission reduction policies might be due to the wider availability of resources in cities with higher GDP [9]. Municipalities (private citizens and industries) with higher availability of resources could be better positioned to invest in low-carbon projects and actions. However, the GDP has been assessed at the provincial level, which may not be representative of the variability of local levels.

The variable delta_MEI_BEI_year has a positive impact on the effectiveness of CO_2_eq emission reduction in cities. Therefore, cities with high values have selected their BEI year between 1990 and 2000. The greater delta between BEI and MEI implies more time to implement mitigation actions, therefore, higher chances to reach the pre-established targets. The greater time availability may also have facilitated the implementation of more actions. In addition, the effectiveness of implemented measures needs a certain time before generating an effective impact on the reduction of emissions. Consequently, cities with BEI further back in time may have included in the SEAPs measures already in place, which, therefore, generated a greater impact in reducing emissions. Together with these factors, technological innovation, progressive increase in attention and awareness to CC mitigation, availability of scientific data, and the emergence of city networks contributing to the exchange of good practices, may have influenced the measures’ effectiveness.

Climatic factors may impact energy consumption, and hence influence CO_2_eq emissions [47]. HDD shows a negative effect on reducing emissions. This is in line with previous literature [10,48,49]. Cities with high HDD values are those with a higher demand for energy needed to heat their buildings.

## 5 Conclusion

This paper analyses the influence of policy mix adopted by CoM cities in reducing their emissions. To perform the assessment, a regression analysis has been applied to 1,354 cities. Results highlight that actions in some sectors, namely *Public lighting*, *Industry*, *Transport*, *Local electricity production*, and *Other*, were effective in reducing cities’ emissions. Furthermore, the policies categorised under *Command and control* class seem to provide effective CO_2_eq reductions. Finally, also the time between BEI and MEI, the population/size, the density, and the GDP are variables that show a positive impact on CO_2_eq emission reduction. On the other hand, from and *Local heat/cold production* sectors, no positive impacts have emerged from the regression analysis. In this case, as described, the following factors play a role in this outcome: i) patterns observed in the building subsectors; ii) typology of policy instruments used to implement actions under these sectors; and iii) measures implementation timeframe. In the future, new monitoring data should be considered and reassessed to evaluate the implementation status and effectiveness of policies adopted in the building and local heat and cold production sectors, which are crucial for reducing emissions and enabling the low-carbon transition in cities.

Moreover, through the analysis, this paper provides insights into the most effective policy instruments that local governments may consider to include in their climate plans. *Command and control* and *Economic* instruments have the capacity to orientate behaviors and promote investments towards low carbon solutions, even if the adoption of some specific instruments might be hampered by political factors. On the contrary, results show that *Information, dissemination, awareness-raising*, and *Other* policy instruments are less effective in reducing CO_2_eq emissions. Considering in particular the former, cities have planned a large number of interventions with a low potential for reduction. This is shown by the 31 % of actions categorised in *Information, dissemination, awareness-raising* instrument, contributing only to 17 % of total expected reductions.

Such results, however, do not suggest that these policy instruments should not be adopted by local governments. Despite difficulties to measure their impact, information and communication actions are key to inform citizens, companies, and other local actors about available opportunities to reduce emissions in their lives and work environment, may lead to behavioural change and may also contribute to building up the necessary skills of local professionals to deliver the energy transition. Furthermore, given the effects on the behaviours, the impact of information and dissemination actions might be more relevant in the long-term and may emerge in monitoring exercises after 2015. Therefore, actions under this category can be complementary and support and reinforce the effectiveness of other types of policies until the longterm outcomes are more defined.

Overall, the results of this study suggest that city's governments should combine several policy instruments and measures in their climate mitigation strategies, as each instrument is characterised by its own strengths and limitations. There is a wide literature on policies for complex environmental challenges, which provides recommendations on how to design effective policy mixes. These include selecting a suitable number of policy instruments and avoiding excessive complexity; selecting instruments that do not conflict with each other and don't lead to contradictory results; provide adequate resources for implementation; and maintain adaptability when designing the instrument [17,50].

Through this study, potential strategies that local governments can include in their plans, taking also in consideration local and territorial circumstances, are highlighted. Therefore, the paper contributes to the literature on local climate policy evaluation, where there is a lack of assessments on the actual results achieved by urban mitigation policies and on the factors that affect emission reduction performances in cities. However, the conducted analysis shows some limitations linked to the dataset setting.

First, the classification of the action into policy instruments is directly performed by public authorities when reporting their data on the platform, which may lead to oversights and inconsistencies. Despite having analysed more than 45,000 actions, this potential bias has not been fully overcome. Secondly, the geographical distribution of signatories in the sample is limited, as the majority of CoM signatories belong to Spain and Italy. Future research should expand the analysis to have a more representative dataset of cities belonging to further countries and, hence, according to data availability, allowing for improving the geographical variability of policies and actions. Finally, some national and regional policies may have relevant impacts on local CO_2_eq emissions, thereby interacting with and influencing the city level performance. Future studies might address these limitations and gaps in developing the dataset. Moreover, future analysis should also rely on updated monitoring data, to expand the cities' and policies' sample and account for the emission reductions achieved by actions that display their effects in the longer term. Finally, the regression model could include an extended group of variables to account for the impact of factors including political priorities of city governments, citizens' and companies’ awareness of CC, the policy mixes adopted by cities in their SEAPs, policy coherence among different levels of government, as well as the impact of European, national and regional climate and energy policies on the effectiveness of local emission reduction performances. Disentangling the contributions of policies at different levels would provide useful insights to policymakers for more effective and consistent policy design in a multi-level perspective.

## Data availability statement

The CoM data are regularly published at https://data.jrc.ec.europa.eu/collection/id-00354.

## Disclaimer

The views expressed are purely those of the authors and may not in any circumstances be regarded as stating an official position of the European Commission.

## CRediT authorship contribution statement

**Benedetta Lucchitta:** Writing - review & editing, Writing - original draft, Methodology, Investigation, Formal analysis, Conceptualization. **Valentina Palermo:** Writing - review & editing, Writing - original draft, Investigation, Formal analysis, Conceptualization, Methodology. **Giulia Melica:** Writing - review & editing, Writing - original draft, Investigation, Formal analysis, Conceptualization, Methodology. **Tania Molteni:** Writing - review & editing, Writing - original draft, Methodology, Investigation, Conceptualization, Formal analysis. **Andrea Burro:** Methodology, Investigation, Writing - original draft. **Paolo Bertoldi:** Writing - review & editing, Supervision, Methodology, Investigation, Conceptualization, Formal analysis. **Edoardo Croci:** Writing - review & editing, Supervision, Methodology, Investigation, Conceptualization, Formal analysis.

## Declaration of competing interest

The authors declare that they have no known competing financial interests or personal relationships that could have appeared to influence the work reported in this paper.

## Figures and Tables

**Figure a:**
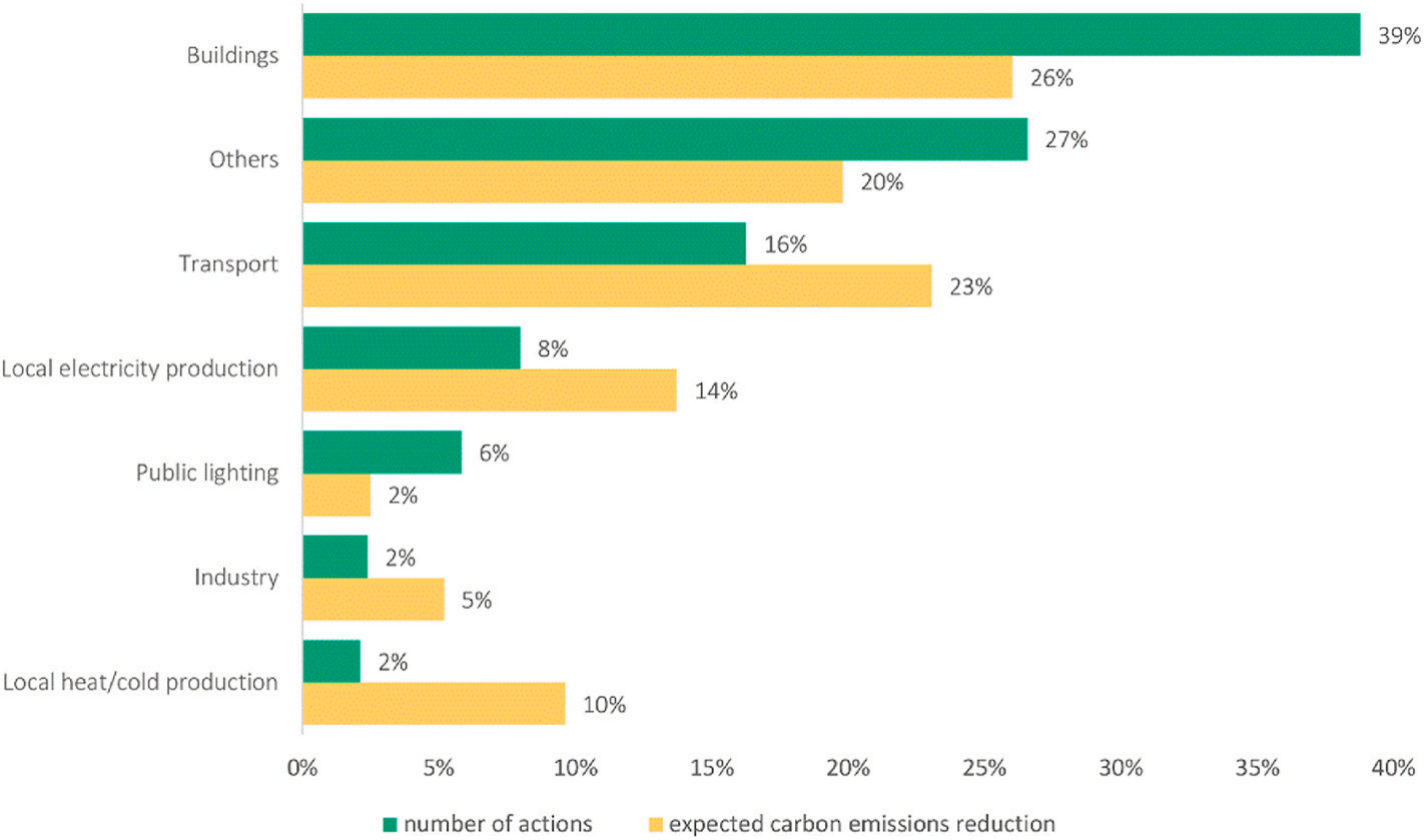
% distributions of the number of mitigation actions and CO_2_eq reductions by sector

**Table a:** Results of the time analysis by sector

Sector	Average of action duration	Share of actions expected to be completed by 2015
Municipal buildings	6.7	25.2 %
Residential buildings	8.0	17.1 %
Tertiary buildings	8.6	10.4 %
Public lighting	6.7	29.8 %
Industry	9.1	9.9 %
Transport	8.0	14.8 %
Local electricity production	7.2	25.3 %
Local heat/cold production	6.4	32.2 %
Others	7.9	17.5 %
**Total**	**7.6**	**19.6 %**

**Table b:** Share of CO_2_eq reduction in planned action in building sub-sectors

Actions belonging to the buildings’ sector, further split by sub-sector	Share on the total planned actions	Share of estimated CO_2_ eq reduction
Municipal buildings	20.0 %	2.7 %
Residential buildings	13.9 %	16.2 %
Tertiary buildings	5.9 %	7.2 %

**Fig. b:**
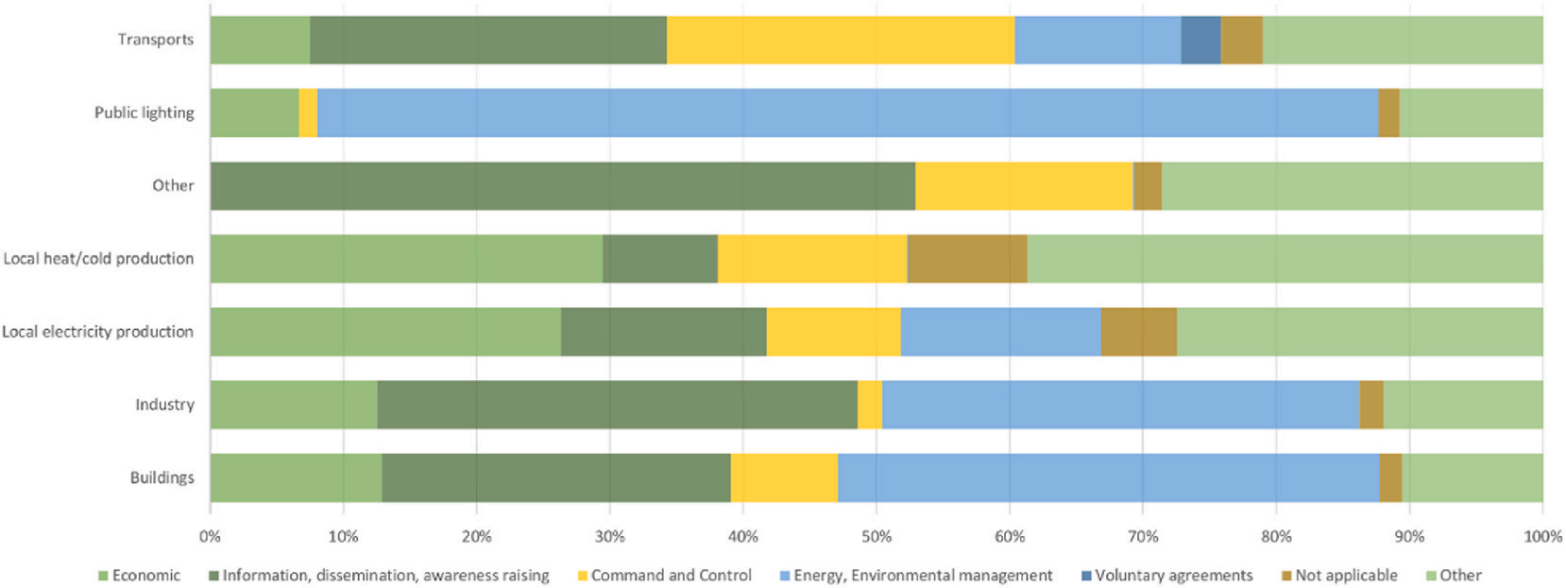
Policy instruments used by sector

**Fig. c:**
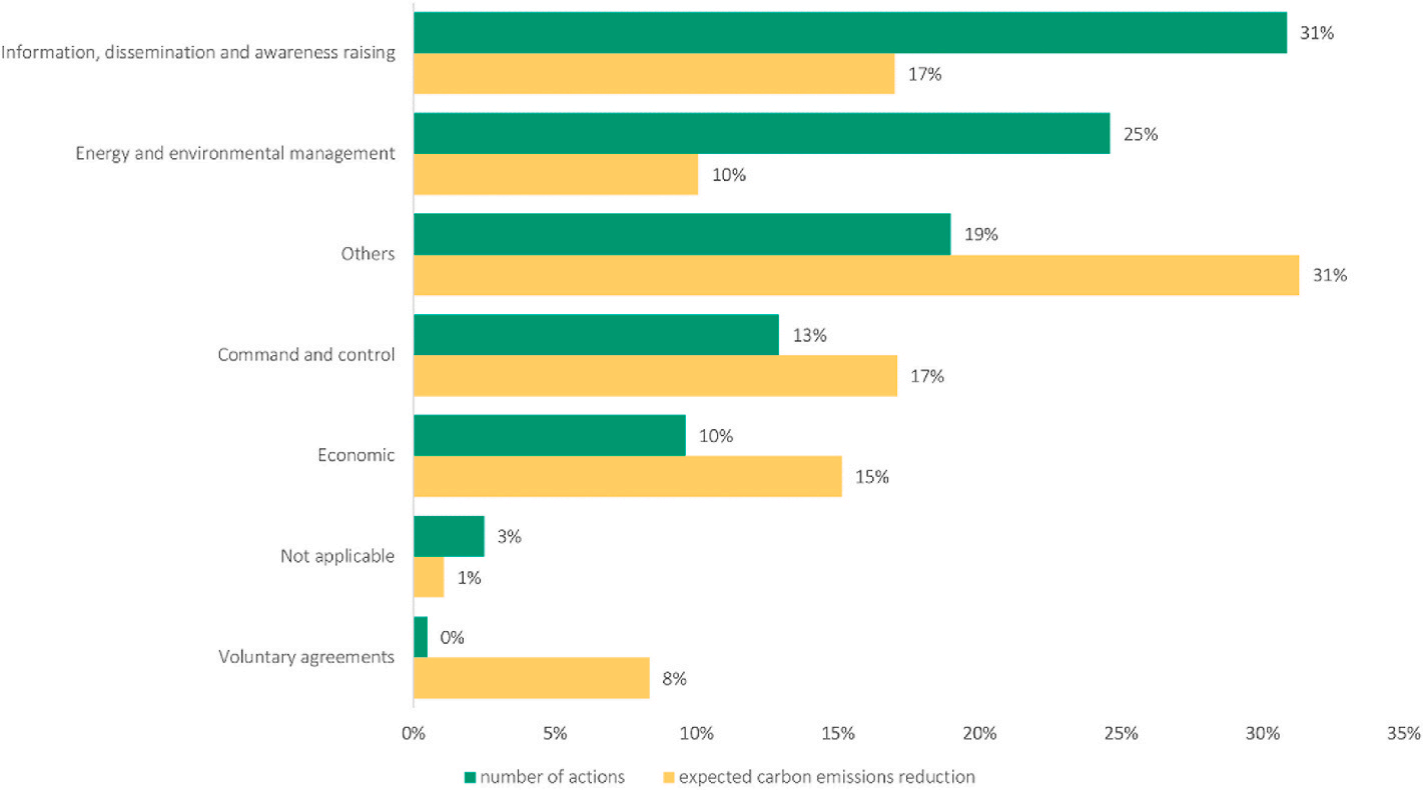
% distributions of the number of mitigation actions and CO_2_eq reductions by category of policy instrument

## Footnotes

2 According to EurVoc classification (https://eur-lex.europa.eu/browse/eurovoc.html?params=72,7206,912#arrow_912).

3 This value is included in the model to test the presence of the Environmental Kuznets Curve [51].

## Footnotes

4 Local Administrative Units - Low level administrative divisions of a country below that of a province, region or state. EUROSTAT, Local Administrative Units (LAU) - NUTS - Nomenclature of territorial units for statistics - Eurostat (europa.eu).

5 The NUTS classification (Nomenclature of territorial units for statistics) is a hierarchical system for dividing up the economic territory of the EU and the UK. NUTS 2 correspond to basic regions for the application of regional policies, NUTS 3 to small regions for specific diagnoses. Available at: Background - NUTS - Nomenclature of territorial units for statistics - Eurostat (europa.eu).

## Footnotes

1 The minimum CO_2_ reduction commitment by 2020 was 20 %.

6 Initially, the waste sector was not included in the reporting framework. As a result, many actions that are currently belonging to the sector “other” and its sub-sectors, were originally recorded elsewhere and here re-assigned.

7 JRC elaborations on ODYSSEE data [52].
